# Synergism of mechanisms underlying early-stage changes in retina function in male hyperglycemic *db/db* mice in the absence and presence of chemically-induced dyslipidemia

**DOI:** 10.1038/s41598-023-44446-3

**Published:** 2023-10-13

**Authors:** Thomas P. Johnston, Genea Edwards, Peter Koulen

**Affiliations:** 1 Department of Ophthalmology, Vision Research Center, School of Medicine, University of Missouri-Kansas City, Kansas City, MO 64108 USA; 2 Division of Pharmacology and Pharmaceutical Sciences, School of Pharmacy, University of Missouri - Kansas City, Kansas City, MO 64108 USA; 3 Department of Biomedical Sciences, School of Medicine, University of Missouri-Kansas City, Kansas City, MO USA

**Keywords:** Endocrine system and metabolic diseases, Preclinical research, Diabetes complications

## Abstract

The study was designed to quantify retina function in a spontaneous mutation mouse model of diabetes, in which sustained dyslipidemia was induced chemically. The goal of the study was to identify if dyslipidemia in the presence of hyperglycemia resulted in either a synergistic, or a merely additive, exacerbation of retinal and visual dysfunctions in diabetes. Two cohorts of mice, male C57BL/6 and C57BL/KsJ-*db/db* mice were divided into two groups each. One group of each strain received the triblock copolymer, poloxamer 407 (P-407), administered by intraperitoneal injection (“WT P-407” and “*db/db* P-407” groups) with saline as a control in the remaining two groups (“WT” and “*db/db*” groups). Blood glucose, total cholesterol (TC) and total triglyceride (TG) levels were quantified using enzyme-based colorimetric assays. Retina function was measured using electroretinography (ERG) and visual acuity was determined by behaviorally assessing parameters of the optomotor reflex. TC and TG levels were normal in both saline controls (WT) and *db/db* mice but were significantly elevated in the WT P-407 group (*p* < 0.01 for TC; *p* < 0.001 for TG), while levels of the same lipids were further elevated in the *db/db* P-407 group when compared to the WT P-407 group levels (*p* < 0.001 for both TC and TG). Behavioral assessment of the optomotor reflex indicated reduced visual acuity for the *db/db* P-407 group when compared to either the WT P-407 or the *db/db* groups (*p* < 0.001, *p* < 0.0001). ERG measurements of scotopic retina function showed a significant decline in the scotopic b-wave amplitude of the WT P-407 animals (*p* < 0.01) and a further reduction for the *db/db* P-407 group when compared to controls (*p* < 0.0001). Very significant, strong correlations between scotopic b-wave amplitude and implicit time to TC (r =  − 0.8376, *p* =  < 0.0001 and r = 0.7069, *p* = 0.0022, respectively) and TG levels (r =  − 0.8554, *p* =  < 0.0001 and r = 0.7150, *p* = 0.0019, respectively) were found. Dyslipidemia in the presence of hyperglycemia synergistically exacerbated the severity of retinal dysfunction in diabetes. P-407 administration significantly elevated plasma TC and TG levels in male wild-type (WT) and diabetic mice (*db/db*), but the resulting hyperlipidemia was more significantly pronounced in the diabetic mice. While elevated plasma lipid and blood glucose levels were individually correlated with a decline in retinal function, the combination of both exacerbated retinal dysfunction. This model of combined hyperglycemia and dyslipidemia can be used to dissect individual contributions of features of the metabolic syndrome to the pathogenesis of retinal dysfunction in diabetes.

## Introduction

Diabetic retinopathy (DR) is a prominent cause of impaired vision in individuals with diabetes secondary to the development of the metabolic syndrome, which is described by the development of hyperglycemia, hypertension, and dyslipidemia^1^. Though DR is classically known as vascular disorder, there is increasing evidence pointing to neuro-retinal dysfunction occurring in early-stage DR, prior to apparent clinical vascular abnormalities^2^. Some of the significant risk factors for the development of DR include race, hypertension, poor glycemic control, and nephropathy, although the duration of diabetes is the greatest risk factor for the development of DR^3,4^. Unfortunately, since the worldwide incidence of diabetes is increasing, vision loss due to DR will become even more prevalent^1^. Importantly, DR is the leading cause of blindness among the workforce (ages 20–74) in developed nations^5^. Based on the current rate of diagnosis of metabolic syndrome, it is estimated that by 2050, possibly up to one-third of the total adult U.S. population could be diagnosed with diabetes, which would result in a massive rise in the number of new occurrences of DR, as well as the healthcare costs associated with DR treatment^6^. For example, the estimated cost for treatment of individuals with DR and its complexities are projected to reach $500 million per year in the United States alone^7,8^. Thus, to establish more effective treatment modalities for DR, the role of distinct aspects of the metabolic disorder in the emergence of DR requires further investigation.

The vast majority of patients with Type 1 diabetes mellitus (T1DM) and approximately 60% of individuals with Type 2 diabetes mellitus (T2DM) display partial signs of DR^9,10^. As previously mentioned, the metabolic syndrome linked with T2DM includes hypertension, hyperglycemia, and dyslipidemia. While the association between hyperglycemia and DR has been relatively well elucidated, this same link has not been unequivocally established between serum lipids and lipoproteins and the development of DR, even though a connection between dyslipidemia and DR was proposed as early as the 1950s^11^. Briefly, lipid profiles from epidemiological studies and the development of DR have proven to be inconsistent. However, current thought linking blood lipids to the development of DR has shifted from attempting to find a correlation between overall lipid profiles and DR to a breakdown of the blood-retinal barrier (BRB) and subsequent extravasation of lipid particles in the retina, which ultimately leads to their oxidation and glycation^12–14^. It has been well documented that these ‘modified’ lipid particles are cytotoxic to all retinal cell types, in addition to capillary cells found in the BR^15–20^. It is also known that DR is not the only eye disease to be affected by modified lipid and lipoprotein particles, since altered local metabolism and peroxidation of lipids have been suggested to play a central role in a number of ocular diseases^21–24^. In the present investigation, we utilized a well-documented and well-established mouse model of dyslipidemia^25–28^ to isolate the contributions that dyslipidemia alone makes to the pathogenesis of DR. Mice are made dyslipidemic with intraperitoneal (i.p.) administration of a triblock copolymer [poloxamer 407 (P-407); avg. MW ~ 12,500 g/mol]^29^. The P-407-induced mouse model of dyslipidemia is able to selectively elevate the two constituents of plasma lipids [triglycerides (TG) and very-low-density lipoprotein cholesterol (VLDL-C)] that are conceivably the most problematic, independent risk factors for DR development^30^, while concurrently producing not only limited levels of high-density lipoprotein cholesterol (HDL-C), but also HDL that is ‘dysfunctional’ with regard to its antioxidant properties^31^. These lipid derangements are significant, since dyslipidemia contributes to the development of DR in over half of afflicted individuals^30^. Additionally, the P-407 mouse model of dyslipidemia creates a cellular oxidative stress environment due to spontaneous and continuous lipid peroxidation. For example, ‘modified’ low-density lipoprotein (LDL)-cholesterol [i.e., oxidized LDL (oxLDL); a known atherogenic stimulus] is produced in this mouse model of dyslipidemia^32^. The P-407 mouse model of induced dyslipidemia was utilized in the following study to examine a number of the phenotypic signature symptoms linked with lipid aberrations described in humans with the metabolic syndrome, which involve low and ‘dysfunctional’ HDL-C, increased TGs, elevated VLDL-C, and the existence of oxLDL. The overarching goal was to define the mechanisms implicated with the pathogenesis of retinal dysfunction stemming from dyslipidemia unaccompanied by additional symptoms of the metabolic syndrome. The use of the *db/db* hyperglycemic mouse model, which is often regarded as an appropriate experimental animal model of T2DM and one that is often utilized to study the development of DR^33,34^, has clinically-insignificant changes in plasma lipids. By studying dyslipidemia alone [P-407-mouse model of induced dyslipidemia with no alterations in blood glucose or insulin], hyperglycemia alone [*db/db* hyperglycemic mouse model with minimal (non-significant) alterations in plasma lipids], and finally, *db/db* hyperglycemic mice made dyslipidemic by treatment with P-407 [simultaneous dyslipidemia and hyperglycemia], permitted a unique approach in which to isolate the effects of dyslipidemia alone, hyperglycemia alone, and both disease-generating mechanisms together on the progression of retinal dysfunction as a factor potentially contributing to the development of DR. Importantly, the approach utilized in the present study allowed for the determination of whether the effects of dyslipidemia and hyperglycemia were either additive, or synergistic, in terms of retinal dysfunction in diabetes.

## Materials and methods

Poloxamer 407 (Pluronic F-127, N.F.) was purchased from Sigma-Aldrich (St. Louis, MO) and used as received. P-407 (i.e., poloxamer 407) is comprised of a hydrophobic central core of poly(oxypropylene) units (MW ~ 4000 g/mol), which is flanked by repeating hydrophilic poly(oxyethylene) groups that terminate in primary hydroxyl groups^29^. The proportions of poly(oxyethylene) and poly(oxypropylene) groups contained in P-407 are 70% and 30%, respectively^29^.

### Animal protocols

Two cohorts of 8-week old, male mice, C57BL/6 (wild type = WT; n = 20) and C57BL/KsJ-*db/db* (n = 20; stock number 000642) were acquired from The Jackson Laboratory (Bar Harbor, ME) and separated into two groups each by random selection. One group (n = 10) of each strain (“WT P-407” and “*db/db* P-407” groups) received P-407 administered by i.p. injection, while saline was used as control in the two remaining groups (“WT” and “*db/db*” groups). Mice were individually housed in plastic shoebox cages at 25 °C and maintained on a 12 h light/dark cycle with ad libitum access to water. Mice were fed Teklad Global 18% Protein Rodent Diet (Envigo, New Jersey; catalog #2918.15) ad libitum except during the 5-h fasting period immediately prior to each blood draw. The institutional animal care and use committee (IACUC) at the University of Missouri-Kansas City (protocol 1503-02) approved all experimental animal procedures and were performed in accordance with institutional and federal guidelines, ARRIVE guidelines and the ARVO Statement for the Use of Animals in Ophthalmic and Vision Research. Additionally, all mice were received at 8 weeks after birth, since this is the time point at which T2DM is firmly established in *db/db* mice. Finally, all mice were provided 1 weeks’ time to acclimate to their environment prior to the following studies.

### Poloxamer 407 (P-407) dosing

Mice contained in the WT P-407 and *db/db* P-407 groups each received an i.p. dose of P-407 dissolved in saline equal to 0.5 g/kg every third day for 30 days (0.5 mL), whereas mice contained in the WT and *db/db* groups each received an i.p. dose of saline only (0.5 mL) every third day for 30 days. The dose of P-407 was periodically adjusted to accommodate increasing body weight as a function of time to ensure a dose of 0.5 g/kg. This was especially important for the *db/db* mice due to rapid weight gain, which normally begins at approximately 4 weeks after birth. Consequently, mice in the *db/db* and *db/db* P-407 groups had a greater initial body weight at 8 weeks after birth (time point when mice were received) than mice contained in the WT and WT P-407 groups, although the rate of weight gain for all groups over the 30-day study was comparable. For example, mice in the *db/db* and *db/db* P-407 groups gained 5.9 g (42.7 ± 2.3 to 48.6 ± 3.6) and 6.2 g (41.3 ± 2.5 to 47.5 ± 3.2) of body weight, respectively, over the 30-day study, whereas mice in the WT and WT P-407 groups gained 6.1 g (23.5 ± 1.8 to 29.6 ± 2.1) and 5.4 g (24.9 ± 1.7 to 30.3 ± 2.6) of body weight, respectively, over the 30-day study.

### Sample collection

Blood samples were collected in plastic capillary tubes from a minimum of 5 mice in each of the four groups prior to initiating experimentation at 9 weeks after birth (acclimation period = 1 week; from week 8 to week 9), and then weekly (weeks 10, 11, 12, and 13 after birth). To obtain a fasted blood sample, mice were denied access to food 5 h prior to tail vein blood collection, but had free access to water. Blood samples were collected, kept on ice, and then processed using a clinical analyzer (described below), with a 5-min run time for complete lipid, lipoprotein, and glucose analysis. All animals were weighed prior to blood collection.

### Quantification of blood lipid, lipoprotein, and glucose concentrations

The blood concentrations of total cholesterol (TC), total TG, HDL, and glucose were determined spectrophotometrically with enzyme-based colorimetric assays using a Cholestek LDX® clinical analyzer (Alere; San Diego, CA). A 40-µl blood sample was placed directly into the well of a cassette, and the cartridge subsequently loaded into the analyzer. Appropriate dilutions were performed for each blood sample if the measured concentration of any analyte fell outside of the instrument’s specified normal linear range, which was especially important for the analysis of TG, since the LDL value is approximated by the Friedewald equation [LDL = TC − HDL − (TG/5)]^35^ and then used by the analyzer to calculate a value for the LDL/HDL ratio. A well-known limitation of the Friedewald equation for estimation of LDL is that it becomes less accurate for TG concentrations above 400 mg/dL, which is why the aforementioned sample dilutions were performed for any analysis resulting in concentration greater than 400 mg/dL. Once the instrument’s software had calculated an LDL concentration, the concentration of VLDL was manually calculated [VLDL = TC − HDL − LDL]. Following estimates of the LDL and VLDL concentrations, the non-HDL concentration was calculated as the sum of LDL and VLDL [i.e., non-HDL = LDL + VLDL]. Finally, to validate the instrument’s measurement of blood glucose, a handheld glucometer (Libre®; Abbott, North Chicago, IL) was used with appropriate reagent strips to obtain a second determination of blood glucose.

### Determination of visual function

Assessment and quantification of visual function using behavioral evaluation previously established in a mouse model of ocular disease was performed using an Optomotry™ system (Cerebral Mechanics, Lethbridge, AB)^36–40^. Visual acuity (VA) is reported as the highest spatial frequency which produces an optomotor response (OMR), defined as an innate, orienting behavior common in animals, fish and insects. Visual acuity was measured at 100% contrast starting at a low spatial frequency (0.042 cycles/degree [c/d]) with gratings alternating rotation between clockwise and anti-clockwise at 12°/s. Contrast sensitivity (CS) is reported as the reciprocal of the contrast threshold value which produces an optomotor response. Contrast sensitivity assessment was performed by reducing the contrast of the alternating gradations while maintaining a constant spatial frequency of 0.042 c/d. A random staircase method was utilized for both tests while the observer was blind to the treatment groups, in addition to spatial frequency or contrast level.

### Electroretinography

Responses from flash electroretinography (ERG) were performed using a Ganzfeld-photostimulator designed for small animals (HMsERG LAB System, Ocuscience LLC, Henderson, NV) and adjunct anesthesia system, following standard procedures adapted for mice^36,37^. Animals were dark-adapted overnight (> 12 h) while all subsequent anesthesia and operational set-up were performed under dim red, dark-adaptive lighting. Each eye received one drop of 0.5% tropicamide 13–20 min to dilate the pupils. Recordings were collected under anesthesia induced with 3.0% isoflurane at an oxygen flow rate of 1 L/min for three minutes, and then maintained at 2.0% with the same flow rate. Animals were placed on a warming pad controlled by a rectal thermometer where core body temperature was maintained at 37 °C. Stainless steel reference and ground subdermal needle electrodes were placed above or below the animal’s ears and near the base of the tail, respectively. GenTeal Tears Gel (0.3% hypromellose) ophthalmologic solution (Alcon, Fort Worth, TX)) was applied to each eye prior to application of silver-embedded thread electrodes. Mini contact lenses (3.0 mm) made from optically clear Aclar material, (Ocuscience LLC, Henderson, NV) filled with 0.3% hypromellose were applied over silver-embedded thread electrodes.

Background activity was recorded for 20 ms prior to each flash and the response was recorded for at least 180 ms afterward. Scotopic flash intensity series (− 5.0 to − 1.5 log cd s/m^2^) was measured with a 1:1,000 neutral density filter (ND3) to control the seven lowest flash intensities. Data was averaged from 49 flashes (− 5.0 to − 4.0 log cd s/m^2^), 10 flashes (− 3.5 log cd s/m^2^), and 4 flashes (− 3.0 to − 1.5 log cd s/m^2^). International Society for Clinical Electrophysiology of Vision (ISCEV) standardized ERG flash protocol, shown in this study as mixed rods and cones, with flash intensity series (− 2.5 to 1.0 log cd s/m^2^) were measured and data averaged from 4 flashes. Data from rods (− 2.5 log cd s/m^2^), Std. (standard) rod-cone response (0.0 log cd s/m^2^), cones with background (BG) (0.0 log cd s/m^2^), Hi-intensity rod-cone response (0.5 log cd s/m^2^), and Hi cones with BG (0.5 log cd s/m^2^) were all averaged from 4 flashes each. Photopic flash intensity series (− 2.0 to 1.5 log cd s/m^2^) was measured and data averaged from 32 flashes on a 30 cd s/m^2^ background after 10 min of light adaptation.

All scotopic, ISCEV, and photopic a- and b-wave amplitudes and implicit times were acquired and processed post-acquisition with ERGView 4.380 V software (Ocuscience LLC, Henderson, NV) with a 60 Hz noise-eliminating filter and 150 Hz low pass filter. Electrode malfunction, electrical, mechanical, and physiological noise may interfere with reliable ERG data so to account for this values that fell 1.5 times outside the upper or lower interquartile range were considered outliers and excluded from the mean. The b/a ratio was determined by ERGView software and provides an index of inner to outer retinal function. Scotopic threshold responses (STRs) and photopic negative responses (PhNRs) were measured from flashes generated from the scotopic flash intensity series and photopic flash intensity series as described previously^36^.

### Statistics

Data are presented as mean ± standard deviation (SD) or standard error of the mean (SEM) where indicated. Analysis of variance using one-way ANOVA with Dunnett’s or Tukey’s post-hoc test was used to determine statistical significance between treatment groups. *p* values < 0.05 were considered statistically significant. The Pearson correlation coefficient (r) was calculated to evaluate strength of relationship between variables. Correlation values were determined as strong for r > 0.6, moderate for r between 0.4 and 0.6, weak for r between 0.2 and 0.4, and no correlation for r < 0.2^36–39^. Statistical analyses and correlations were achieved using GraphPad Prism 8.0 software (GraphPad Software Inc., La Jolla, CA). To maintain consistency for different levels of statistical significance (p values) for all of the figures and tables included in the present study, we have elected to use the following symbols; *p < 0.05, **p < 0.01, ***p < 0.001, and ^#^*p* < 0.0001.

## Results

### Blood glucose, lipid levels, and lipoprotein fractions in dyslipidemia and/or hyperglycemia

**Figure 1:**
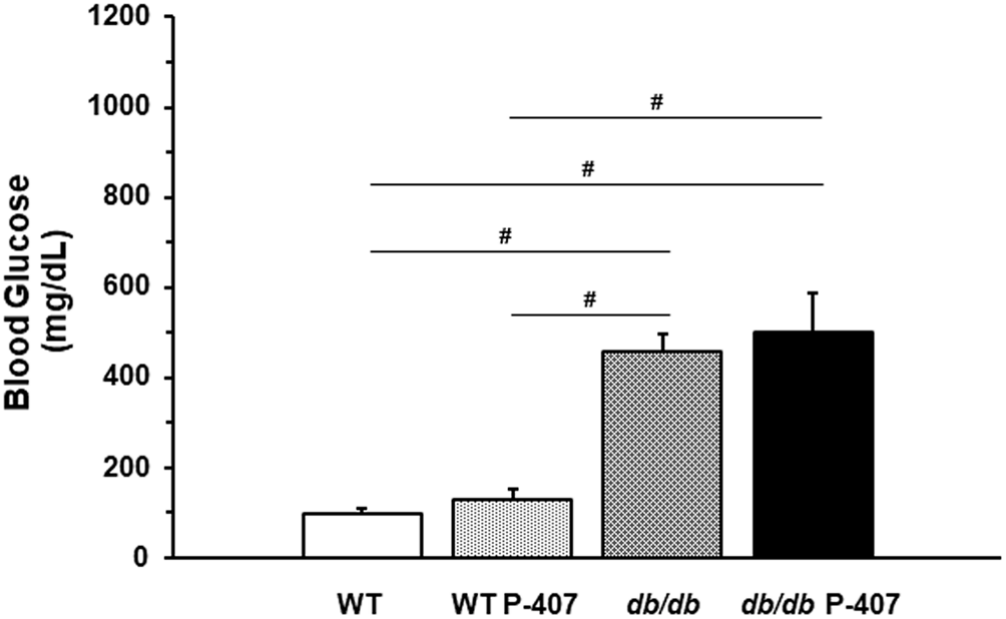
The effect of diabetes spontaneous mutation on blood glucose levels in the absence and presence of poloxamer 407-induced dyslipidemia. Each mean value depicted in the graph represents the average blood glucose concentration of the blood samples collected at weeks 10, 11, 12, and 13 for each group of mice. Values are the mean ± SD of 5 mice from each group. ^#^ designates a statistically significant difference (*p* < 0.0001) between mean values.

As shown in Fig. 1, P-407 does not significantly (*p* > 0.05) perturb blood glucose levels, whether used alone in wild-type mice (WT vs. WT P-407), or diabetic mice (*db/db* vs. *db/db* P-407). However, as expected, hyperglycemia caused by a spontaneous mutation was significantly (*p* < 0.0001) elevated in the *db/db* strain relative to WT mice. Importantly, while it has previously been shown that P-407 does not alter blood glucose levels when administered to WT mice^41^, it also appears to have no additional hyperglycemic effect when administered to *db/db* mice (Fig. 1; bar 3 vs. bar 4).

**Figure 2:**
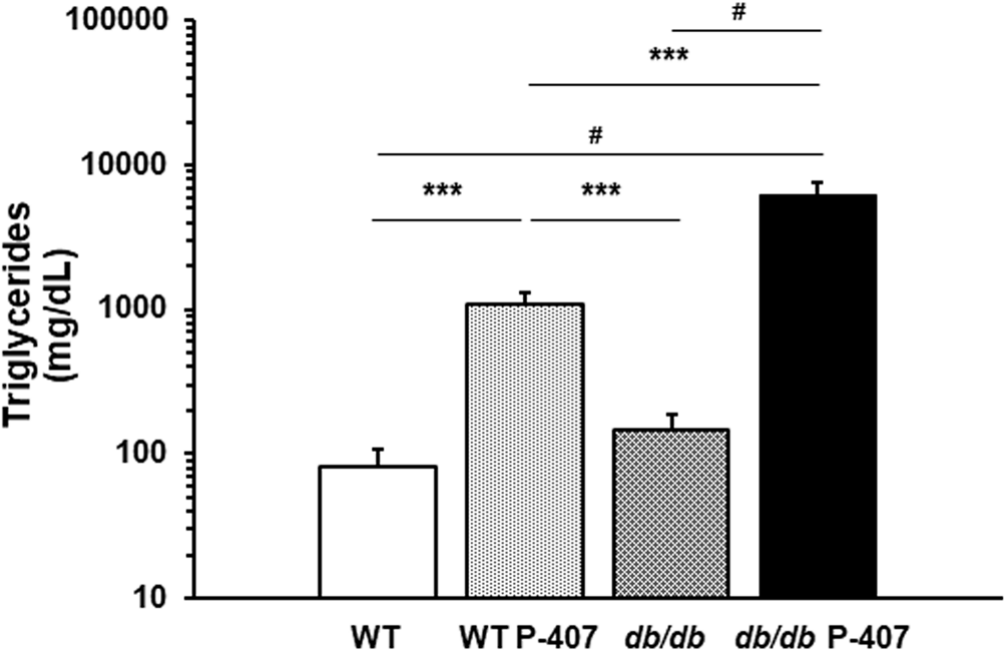
The effect of diabetes spontaneous mutation on plasma triglyceride levels in the absence and presence of poloxamer 407-induced dyslipidemia. Each mean value depicted in the graph represents the average blood glucose concentration of the blood samples collected at weeks 10, 11, 12, and 13 for each group of mice. Values are the mean ± SD of 5 mice from each group. *** and ^#^ designate a statistically significant difference (*p* < 0.001 and *p* < 0.0001, respectively) between mean values.

**Figure 3:**
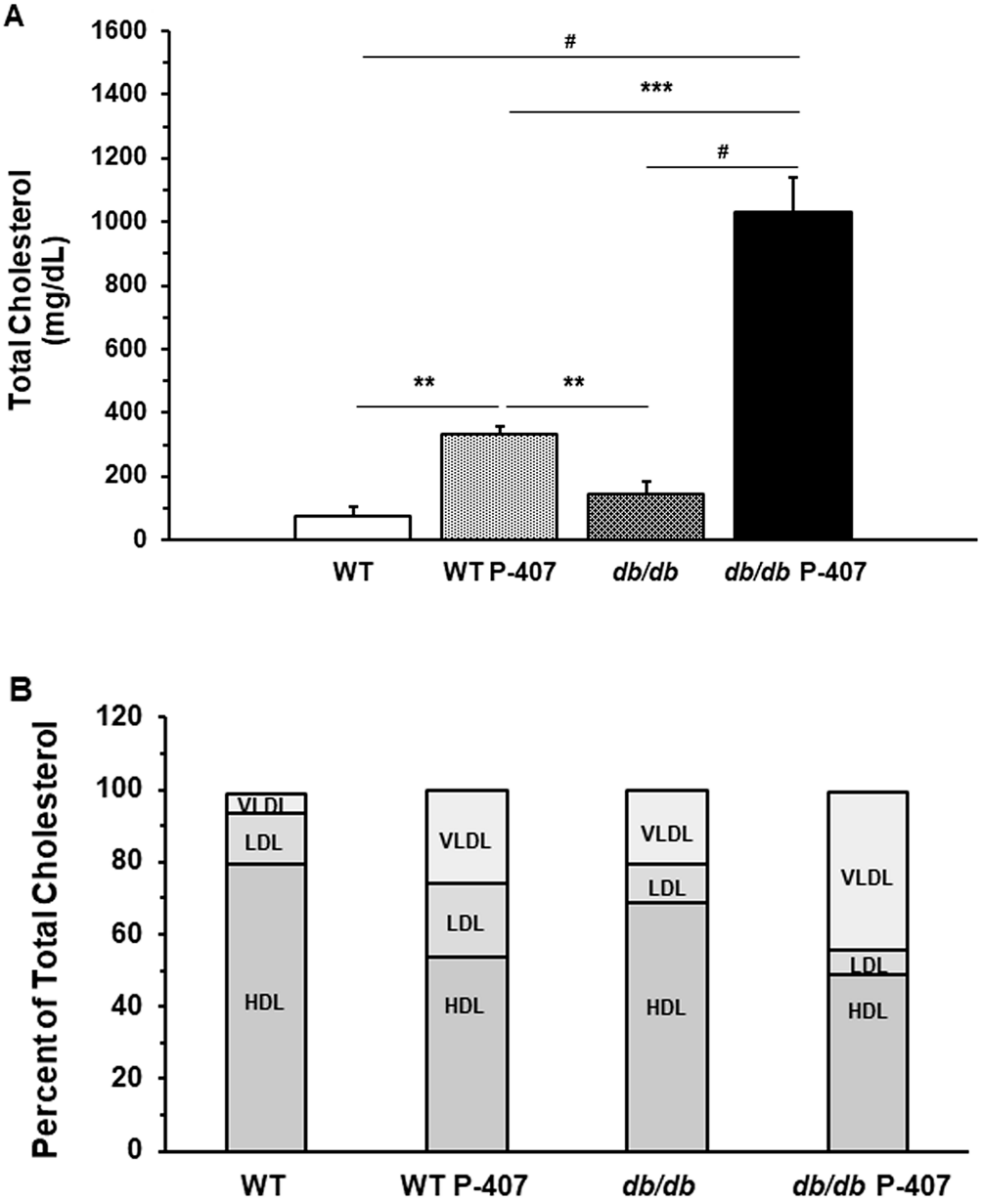
The effect of diabetes spontaneous mutation on plasma total cholesterol levels and plasma lipoprotein concentrations. Plasma total cholesterol levels (**A**) in the absence and presence of poloxamer 407-induced dyslipidemia. Each mean value depicted in the graph in 3A represents the average blood glucose concentration of the blood samples collected at weeks 10, 11, 12, and 13 for each group of mice. Values are the mean ± SD of 5 mice from each group. **, ***, and ^#^ designate a statistically significant difference (*p* < 0.01, *p* < 0.001, and *p* < 0.0001, respectively) between mean values. Plasma lipoprotein concentrations (**B**) are shown as a percent of the plasma concentration of total cholesterol for diabetes spontaneous mutation (*db/db*) and WT mice in the absence and presence of poloxamer 407-induced dyslipidemia. The percent of each plasma lipoprotein fraction for each group of mice (n = 5) represents a mean value. Levels of statistical significance for each lipoprotein fraction for each group of mice (p values) are tabulated in Supplemental Table S1.

A profound elevation in TGs, relative to TC, was detected in the P-407-treated groups, since P-407 is a non-specific lipase inhibitor in vivo and specifically inhibits lipoprotein lipase (Fig. 2 vs. Figure 3A)^42,43^. Figure 2 also shows the effect of diabetes spontaneous mutation (*db/db*) on plasma TG levels in the absence and presence of dyslipidemia induced by administration of P-407 (Fig. 2; bar 3 vs. bar 4).

The *db/db* mouse model displays minimal, clinically non-significant perturbations in blood lipids and lipoproteins^44,45^. We were able to confirm this, since the mean value of the TG concentration in the *db/db* mice was not statistically different from that in WT mice (Fig. 2; bar 1 vs. bar 3). The administration of P-407 to WT mice significantly (*p* < 0.001) increased the TG concentration when individually compared to either WT or *db/db* mice (Fig. 2). However, the P-407-mediated elevation in TG was even more significant when P-407 was administered to *db/db* mice, with TG levels being significantly (*p* < 0.001) greater relative to TG in WT P-407 mice (Fig. 2; bar 2 vs. bar 4), and profoundly (*p* < 0.0001) elevated when individually compared to either WT (Fig. 2; bar 1 vs. bar 4) or *db/db* (Fig. 2; bar 3 vs. bar 4) mice.

Changes in TC are shown in Fig. 3A. TC levels, similar to TG levels shown in Fig. 2, were not significantly different between WT and *db/db* mice. While the TC concentration was significantly (*p* < 0.01) greater in P-407-treated WT mice when individually compared to corresponding TC concentrations determined in WT (Fig. 3A; bar 1 vs. bar 2) and *db/db* (Fig. 3A; bar 2 vs. bar 3) mice, it was profoundly (*p* < 0.0001) elevated in *db/db* P-407 mice when individually compared to TC concentrations in WT (Fig. 3A; bar 1 vs. bar 4) and *db/db* (Fig. 3A; bar 3 vs. bar 4) mice. Interestingly, *db/db* mice exhibited a much greater response (increase in TC) than WT mice when administered an equivalent dose of P-407 [Fig. 3A; WT P-407 (bar 2) vs. *db/db* P-407 (bar 4); *p* < 0.001].

More informative was how the individual lipoproteins that comprise TC are altered in both WT and *db/db* mice in the absence, or presence, of P-407-induced dyslipidemia. Figure 3B depicts each lipoprotein as a percent of TC, which assumes that TC = HDL + LDL + VLDL [the concentration of intermediate-density lipoprotein (IDL) was not determined]. As it pertains to *db/db* mice, diabetes spontaneous mutation resulted in a percent lipoprotein composition of TC that was in fairly close agreement to that determined for WT mice, with a slight reduction in both HDL (n.b., ~ 80% HDL is considered normal in WT mice) and LDL, but an increase in VLDL (Fig. 3B; bar 1 vs. bar 3; Table S1).

Some of the most prominent features in the fractional lipoprotein composition of TC were observed in WT mice treated with the general lipase inhibitor P-407 (WT P-407), which produced a profound reduction in the percent HDL (~ 30% reduction; from 79.4 to 53.5%), a marked increase in the LDL fraction (~ 6% increase; from 14.2 to 20.5%), and a significant expansion in the VLDL fraction (~ fivefold greater; from 5.4 to 25.6%) when compared to these same lipoprotein fractions in WT mice (Fig. 3B; bar 1 vs. bar 2; Table S1). When the HDL and VLDL fractions in *db/db* mice were compared to the corresponding HDL and VLDL fractions determined for *db/db* P-407 mice, these same trends of a significant reduction in the percent HDL (~ 20% decrease; from 68.8 to 49.0%), and especially an expanded VLDL fraction (~ 25% increase; from 20.6 to 44.0%) were also observed (Fig. 3B; bar 3 vs. bar 4; Table S1).

### Reduced visual function in dyslipidemia and hyperglycemia

**Figure 4:**
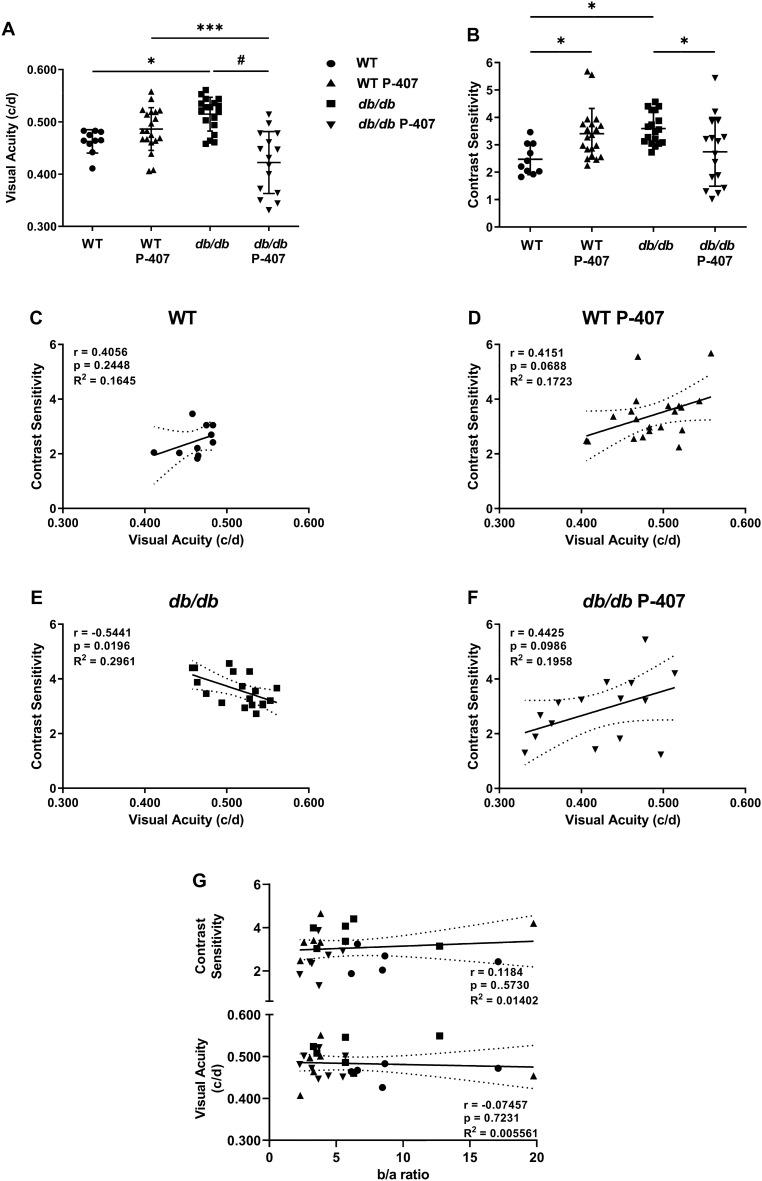
Visual function in the absence and presence of poloxamer 407-induced dyslipidemia. Behavioral assessment of quantitative measures of the optomotor reflex for visual acuity (**A**) represented as spatial frequency (in c/d), and contrast sensitivity (**B**) for WT control (n = 5) (circles), WT P-407 (n = 10) ( (triangles), *db/db* (n = 6) (squares), and *db/db* P-407 (n = 10) (inverted triangles). Visual acuity was correlated with contrast sensitivity individually for all four groups of mice, WT (**C**), WT P-407 (**D**), *db/db* (**E**), and *db/db* P-407 (**F**). Visual acuity and contrast sensitivity was also correlated with ERG b/a wave ratio (**G**). Pearson correlation coefficient r, *p* values, and goodness of fit R^2^ are listed directly in panels. Data are presented as mean ± SD. One-way ANOVA with Tukey’s post-hoc test was used to determine statistical significance of mean values for each group of mice. *, ***, and ^#^ designate a statistically significant difference (*p* < 0.05, *p* < 0.001, *p* < 0.0001, respectively) between mean values.

Visual function was quantified by behavioral assessment of the optomotor reflex (Fig. 4). All four cohorts of mice had VA and CS measured at the beginning (data not shown) and conclusion of the study. Final VA measurements in *db/db* mice treated with P-407 were significantly reduced when compared to the WT mice treated with P-407 and *db/db* mice groups (Fig. 4A). Final CS measurements were also reduced in the *db/db* P-407 group (*p* < 0.05) compared to the *db/db* group (Fig. 4B). Scatter plot analysis by the Pearson correlation coefficient calculation shows VA correlates positively and moderately (r = 0.4056, *p* = 0.2448) with CS in WT animals (Fig. 4C), WT P-407 animals (r = 0.4151, *p* = 0.0688) (Fig. 4D), and with *db/db* P-407 animals (r = 0.4425, *p* = 0.0986) (Fig. 4F), while VA correlates negatively and moderately (r =  − 0.5441, *p* = 0.0196) (Fig. 4E) with CS in *db/db* animals indicating that the close linear relationship between VA and CS is maintained during visual impairment caused by dyslipidemia and hyperglycemia. No correlation was found between the ERG b/a ratio and VA or CS (Fig. 4G).

### Relationship between blood glucose and plasma lipid levels to visual function

**Figure 5:**
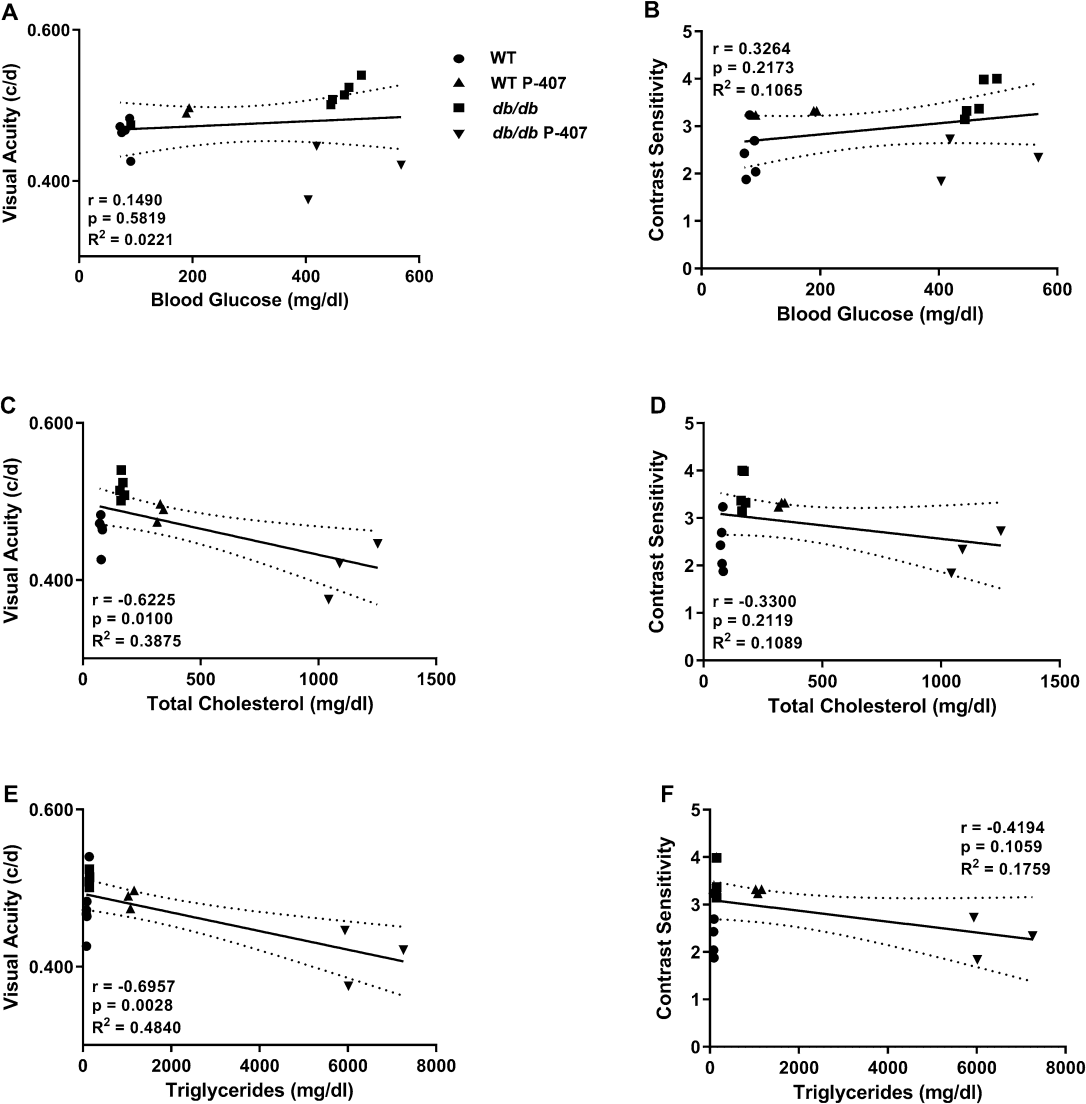
Relationship between blood glucose and plasma lipid levels to visual function. Correlations were determined between behavior assessment of visual function and blood glucose (**A**,**B**), total cholesterol (**C**,**D**), and plasma triglycerides (**E**,**F**). WT controls (n = 10) are symbolized with circles, WT P-407 (n = 7) with triangles, *db/db* (n = 7) with squares, and *db/db* P-407 (n = 6) with inverted triangles. Dashed lines around the regression line indicate 95% CI. Specific Pearson correlation coefficient r, respective *p* values, and coefficient of determination R^2^ are listed directly in panels.

We also examined the relationship between blood glucose and plasma lipid levels to behavioral-assessed visual function in Fig. 5. VA presented no measurable correlation to blood glucose levels, while CS showed a positive, but weak correlation (r = 0.3264, *p* = 0.2173) that was not statistically significant (Fig. 5A,B, respectively). TC and TG both demonstrated a significantly strong, negative correlation (r =  − 0.6225, *p* = 0.0100 and r =  − 0.6957, *p* = 0.0028, respectively) with VA (Fig. 5C,E). However, TC and TG showed only a weak to moderate negative correlation (r =  − 0.3300 and r =  − 0.4194, respectively) to CS (Fig. 5D,F) that did not reach significance.

### Decreased inner retinal function in dyslipidemic and hyperglycemic animals

**Figure 6:**
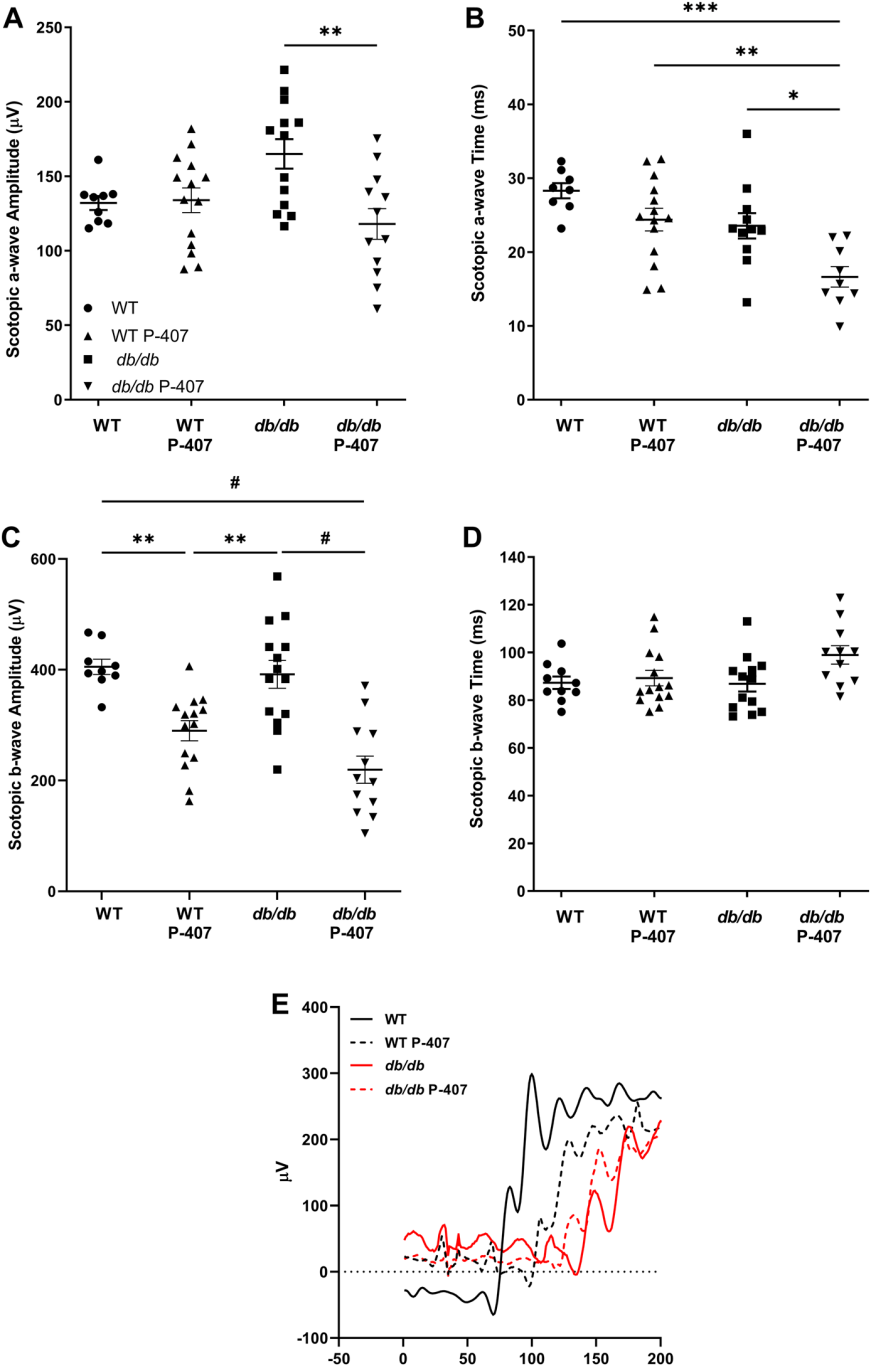
Retinal function in the absence and presence of poloxamer 407-induced dyslipidemia. Full-field flash ERG was utilized to assess scotopic (**A**–**E**) function. WT controls (n = 10) are indicated with circles, WT P-407 (n = 7) with triangles, *db/db* (n = 7) with squares, and *db/db* P-407 (n = 6) with inverted triangles. A representative trace from a single eye out of each group is presented in (**E**). Solid black and red lines indicate WT animals and *db/db* animals respectively, while dashed lines indicate animals treated with P-407. Data are presented as mean ± SEM for each group of mice. One-way ANOVA with Tukey’s post-hoc test was used to determine statistical significance. *, **, ***, and ^#^ designate a statistically significant difference (*p* < 0.05, *p* < 0.01, *p* < 0.001, and *p* < 0.0001, respectively) between mean values.

Full-field flash ERG measurements assessing scotopic, mixed rods and cones (ISCEV), and photopic function were recorded for all four cohorts of mice (Figs. 6, S1, S2). ERG measurements of scotopic function indicated a significant (*p* < 0.01) decline in the a-wave amplitude with the addition of P-407 to *db/db* mice compared to *db/db* mice (Fig. 6A). There was also a significant (0.05 < *p* < 0.01 *p* < 0.001) reduction in the implicit times of the a-wave with the *db/db* P-407 animals compared to the *db/db*, WT P-407, and WT mice, respectively (Fig. 6B). A significant (*p* < 0.01) decline in the b-wave amplitudes of the WT P-407 animals compared to WT mice was observed, while an even more significant (*p* < 0.0001) decline in the b-wave amplitudes were noted for the *db/db* P-407 group compared to the *db/db* animals. The b-wave function of the WT-P407 mice was significantly reduced when compared to that of *db/db* mice (*p* < 0.01). The *db/db* P-407 group also showed a very significant (*p* < 0.0001) decline in the b-wave amplitude compared to WT mice (Fig. 6C). There were no significant delays in implicit time for the scotopic b-wave function (Fig. 6D). A single representative scotopic trace is shown at 1.5 log cd s/m^2^ for all four groups of mice (Fig. 6E).

Responses for mixed rod and cone function (ISCEV protocol) showed a significant (*p* < 0.05) increase in the amplitude of a- and b-wave for the *db/db* group compared to WT P-407 animals (Fig. S1A,C). A significant (*p* < 0.05) decline in amplitude was observed in the a-wave with the addition of P-407 to the *db/db* group relative to *db/db* animals (Fig. S1A). The WT P-407 group exhibited a significant (*p* < 0.05) increase in the a-wave implicit time when compared to WT animals. Additionally, the *db/db* animals displayed a significant (*p* < 0.05) decrease in a-wave implicit time compared to WT P-407 animals, while the *db/db* P-407 group had an even more significant (*p* < 0.01) decrease in the a-wave implicit time compared to the WT P-407 group (Fig. S1B). The *db/db* animals showed a significant (p < than 0.05) increase in b-wave amplitude compared to WT P-407 animals (Fig. S1C). Significant (*p* < 0.05) delays were found for the b-wave implicit time with the WT P-407 animals relative to the WT and *db/db* groups (Fig. S1D). A single representative mixed rod and cone trace is shown at 1.0 log cd s/m^2^ for all four groups of mice (Fig. S1E).

Responses for photopic function showed a significant (*p* < 0.05) decrease in the a-wave implicit time observed in *db/db* P-407 mice when compared to the WT P-407 group (Fig. S1G). Lastly, a significant (0.05 < *p* < 0.01) increase in the amplitude of the photopic b-wave was noted in the *db/db* group of mice compared to both groups that were made hyperlipidemic, WT P-407 and *db/db* P-407, respectively (Fig. S1H). A single representative photopic trace is shown at 1.5 log cd s/m^2^ for all four groups of mice (Fig. S1J).

### Impaired scotopic and photopic retinal function

Flashes for scotopic a-wave amplitudes (Fig. S2A, Table S2) show deficits in flashes beginning at − 5.5 to − 2.5 log cd s/m^2^ for WT-P407 and *db/db* animals with more significant deficits from the d*b/db* P-407 group from − 5.0 to 0.0 log cd s/m^2^. ERG b-wave scotopic amplitudes displayed significant differences for the *db/db* mice and both P-407 groups. The WT P-407 mice displayed deficits in flashes beginning at − 5.5 to 1.0 log cd s/m^2^ but more significantly for the *db/db* P-407 mice, most notably from − 3.0 to 1.0 log cd s/m^2^ (Fig. S2C, Table S2). Differences in scotopic a-wave implicit time (Fig. S2B, Table S2) were significant for the *db/db* P-407 group from − 5.5 to 0.0 log cd s/m^2^ and for the b-wave implicit time (Fig. S2D, Table S2), where differences were also significant, most notably from − 2.5 to 0.5 log cd s/m^2^. Flashes for the photopic a- and b- wave amplitudes (Fig. S2E–G, Table S2) and implicit times (Fig. S2F,H, Table S2) showed deficits in the WT P-407 and *db/db* P-407 groups beginning at − 2.5 to 1.0 log cd s/m^2^. The photopic a-wave amplitude (Fig. S2E, Table S2) for the *db/db* P-407 group of animals only displayed a significant difference in amplitude early at − 2.5 log cd s/m^2^ while the photopic b-wave amplitude (Fig. S2G, Table S2) for the WT P-407 animals displayed differences between 0.0 to 1.0 log cd s/m^2^. Significant deficits in a- and b-wave implicit times (Fig. S2F,H, Table S2) were also noted between − 0.5 to 1.0 for the WT animals treated with P-407.

### Changes in threshold sensitivity of scotopic and photopic responses

In response to flash intensities below − 3.6 log cd s/m^2^, where STRs signify inner retina function^46^, *db/db* P-407 mice had significantly reduced positive STR (pSTR) (Fig. S3A) beginning at − 4.5 log cd s/m^2^ (*p* = 0.0111) and − 4.0 log cd s/m^2^ (*p* = 0.0488) compared to the *db/db* group. The *db/db* P-407 mice also showed a significant difference of the pSTR at − 3.0 log cd s/m^2^ (*p* = 0.0242) compared to WT animals. The negative STR (nSTR) (Fig. S3B) displayed no significant difference between cohorts. There was a significant difference in the STR amplitude (Fig. S3C) beginning at − 4.5 log cd s/m^2^ of the WT P-407 group (*p* = 0.0237 and *p* = 0.0120) compared to WT animals. STR amplitude implicit time (Fig. S3D) also showed a significant difference at − 3.5 log cd s/m^2^ for the *db/db* animals (*p* = 0.0324) compared to WT mice. While the PhNRs (Fig. S3E), which originate from RGCs and their axons^47^, were not significantly different between cohorts, PhNR implicit time (Fig. S3F) exhibited a significant difference at 0.0 log cd s/m^2^ for the *db/db* P-407 animals (*p* = 0.0244) compared to the mice in the WT P-407 group and 0.5 log cd s/m^2^ for the *db/db* group (*p* = 0.0244) compared to WT animals.

### Relationship between retinal function and behavior assessment of optomotor reflex

**Figure 7:**
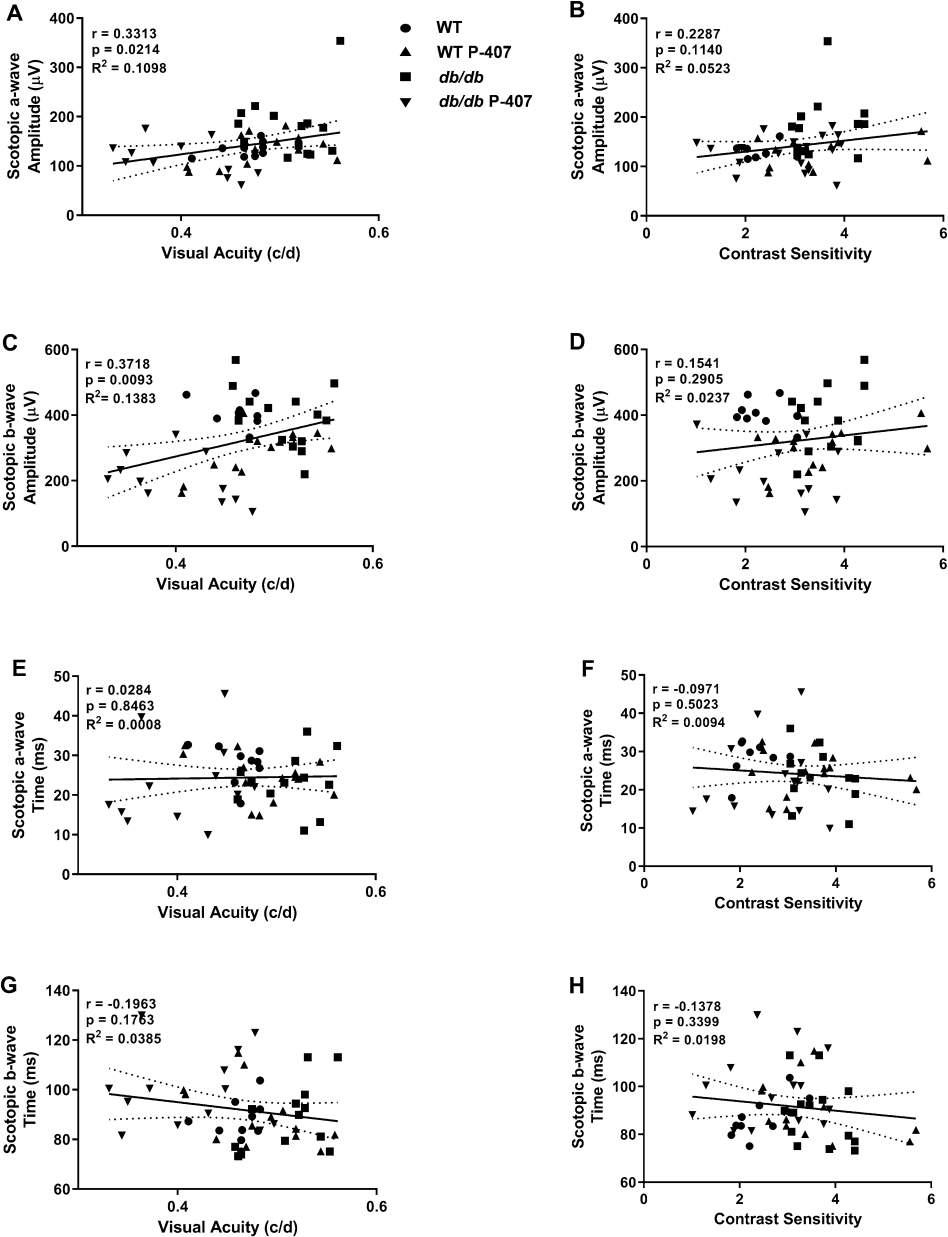
Relationship between scotopic ERG function and behavior assessment of optomotor reflex. Behavioral assessment of the optomotor reflex was correlated with scotopic ERG amplitude (**A**–**D**) and implicit time (**E**–**H**). WT controls (n = 10) are symbolized with circles, WT P-407 (n = 7) with triangles, *db/db* (n = 7) with squares, and *db/db* P-407 (n = 6) with inverted triangles. Dashed lines around the regression line indicate 95% CI. Specific Pearson correlation coefficient r, respective *p* values, and coefficient of determination R^2^ are listed directly in panels.

Relationships between visual function variables and retinal function variables made up of components of ERG responses allow us to determine an association, extent, and direction of the relationship between the variables. The a-wave reflects the overall general health of the photoreceptors found in the outer retina while the b- wave reflects the overall health of the inner retina which includes ON bipolar and Müller cell activity. Correlations between VA, CS, and Scotopic ERG responses were identified for all four cohorts of mice (Fig. 7). VA correlates weakly, yet significantly (r = 0.3313, *p* = 0.0214), with scotopic a-wave amplitude and very significantly (r = 0.3718, *p* = 0.0093) with b-wave amplitude (Fig. 7A,C). Contrast sensitivity also correlates weakly, but not significantly (r = 0.2287, *p* = 0.1140), with scotopic a-wave (Fig. 7B). Moreover, CS and scotopic b-wave (Fig. 7D) showed no correlation, nor did VA and CS, to implicit time (Fig. 7E–H). Finally, no relationship between mixed rod and cone ERG function and optomotor reflex assessment of VA and CS was found (Fig. S4A–H), nor was a relationship identified between photopic ERG function and optomotor reflex (Fig. S5A–F,H). However, a weak correlation was found between VA and photopic b-wave implicit time (Fig. S5G), although it did not achieve statistical significance. We can conclude from these correlations that there is a weak association between decreased visual function (VA and CS) with scotopic (low lit conditions) a- and b- wave amplitudes. There is also a weak association between VA and photopic (well-lit conditions) b-wave time (stimulus onset to peak of the b-wave) when both WT and *db/db* animals are made hyperlipidemic with P-407.

### ERG b/a wave ratios

**Figure 8:**
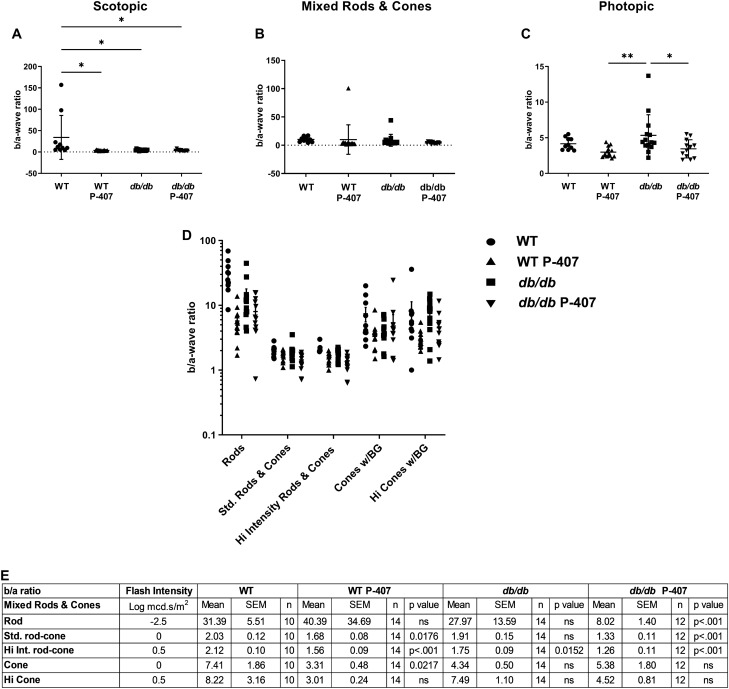
ERG b/a wave ratio. The b/a wave ratios of amplitudes for all 4 cohorts of animals were calculated for scotopic (**A**), mixed rod and cone (**B**), and photopic function (**C**) for five specific responses (**D**). WT controls (n = 10) are symbolized with circles, WT P-407 (n = 7) with triangles, *db/db* (n = 7) with squares, and *db/db* P-407 (n = 6) with inverted triangles. Statistical values from (**D**, **E**). Data are presented as mean ± SD (**A**–**C**) and ± SEM (**D**,**E**). One-way ANOVA with Tukey’s post-hoc test was used to determine statistical significance for each group of mice. * and ** designate a statistically significant difference (*p* < 0.05, and *p* < 0.01, respectively) between mean values.

Analysis of ERG data is not only based on amplitude and implicit time but also the relationship between the a- and b-waves. The a-wave reflects activity in the photoreceptors found in the outer retina while the b-wave originates from the Müller and bipolar cells found in the inner retina. The b/a ratio reflects a signal transmission relationship between the two components. The b/a ratio for normal mice is characteristically greater than 1.6^48^, with WT C57Bl/6 mice generally displaying b/a ratios of 1.9–2.4^49^. The scotopic b/a wave ratio of the WT P-407, *db/db*, and *db/db* P-407 groups were all statistically significantly different when individually compared to the scotopic b/a wave ratio for the WT mice (Fig. 8A). There were no significant differences found between mixed rods and cones and b/a ratios (Fig. 8B). Photopic b/a wave ratios displayed a very significant (*p* < 0.01) difference in the *db/db* group compared to the WT P-407 animals and a significant (*p* < 0.05) difference in the *db/db* P-407 group compared to the d*b/db* group (Fig. 8C). The b/a wave ratios for the five specific ISCEV responses (Fig. 8D) are graphed, and statistical analyses are presented in the accompanying table (Fig. 8E). WT animals made hyperlipidemic with P-407 had statistical differences in Std. rod-cone b/a ratios (1.68 ± 0.08, *p* = 0.0176), Hi Int. rod-cone (1.56 ± 0.09, *p* < 0.001, and cone (3.31, ± 0.48, *p* = 0.0217) compared to the WT group (2.03 ± 0.12), (2.12 ± 0.10), and (7.41 ± 1.86), respectively. Diabetic animals made hyperlipidemic with P-407 had statistical differences in rod b/a ratios (8.02 ± 1.40, *p* < 0.001), Std. rod-cone (1.33 ± 0.11, *p* < 0.001), and Hi Int. rod-cone (1.26 ± 0.11, *p* < 0.001) compared to the WT group (31.39 ± 5.51), (2.03 ± 0.12), and (2.12 ± 0.10), respectively. The observed b/a ratios for four specific ISCEV responses (rod, Std. rod-cone, Hi Int. rod-cone, and cone) show that hyperlipidemia significantly affects both rods and cones, which can be separated out for their individual contribution to the ISCEV ERG protocol assessed under dark-adapted and light-adapted conditions.

### Decreased inner retinal function correlates with blood glucose and elevated plasma lipids

**Figure 9:**
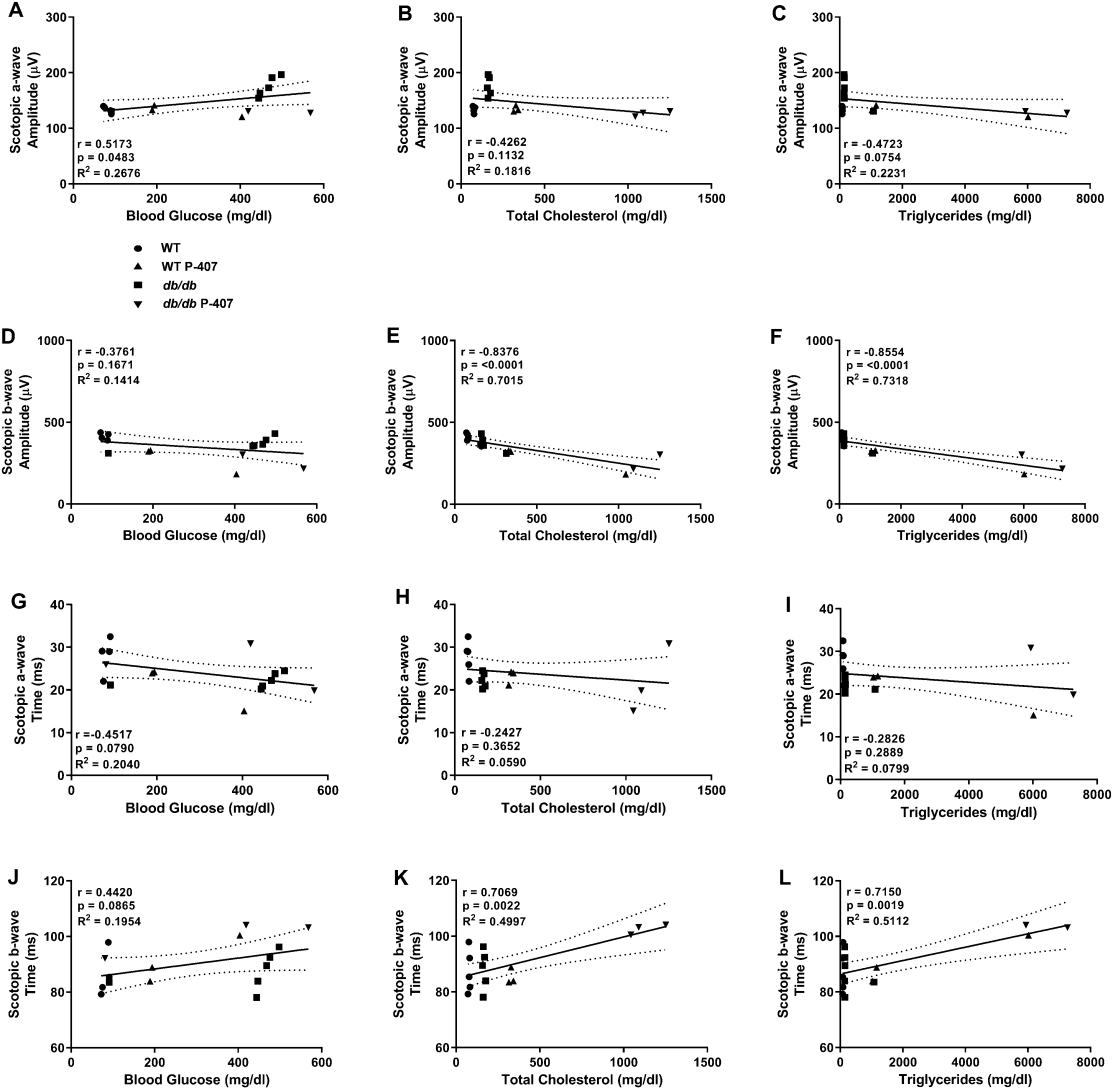
Relationship between blood glucose, plasma lipid levels, and scotopic ERG function. Correlations were determined between scotopic a- and b-wave amplitudes, implicit time, and blood glucose (**A**,**D**,**G**,**J**), total cholesterol (**B**,**E**,**H**,**K**), and plasma triglycerides (**C**,**F**,**I**,**L**). WT controls (n = 10) are symbolized with circles, WT P-407 (n = 7) with triangles, *db/db* (n = 7) with squares, and *db/db* P-407 (n = 6) with inverted triangles. Dashed lines around the regression line indicate 95% CI. Specific Pearson correlation coefficient r, respective *p* values, and coefficient of determination R^2^ are listed directly in panels.

Scatter plot analysis showed blood glucose had a moderate to weak, statistically significant positive and negative correlation to scotopic a- and b-wave amplitudes (Fig. 9A,D) (r = 0.5173, *p* = 0.0483 and r =  − 0.3761, *p* = 0.1671, respectively). Additionally, blood glucose also had moderate, negative and positive correlations to scotopic a-wave and b-wave implicit time (Fig. 9G,J) (r =  − 0.4517, *p* = 0.0790, and r = 0.4420, *p* = 0.0865, respectively), though not significant. Moderate to weak, statistically insignificant, negative correlations were found between scotopic a-wave amplitude (Fig. 9B) (r =  − 0.4262, *p* = 0.1132) and implicit time (Fig. 9H) (r =  − 0.2427, *p* = 0.3652) for TC and between scotopic a-wave amplitude (Fig. 9C) (r =  − 0.4723, *p* = 0.0754) and implicit time (Fig. 9I) (r =  − 0.2826, *p* = 0.2889) for TG. Strongly, negative and positive correlations were seen between scotopic b-wave amplitude (Fig. 9E) (r =  − 0.8376, *p* =  < 0.0001) and implicit time (Fig. 9K) (r = 0.7069, *p* = 0.0022) for TC and between scotopic b-wave amplitude (Fig. 9F) (r =  − 0.8554, *p* =  < 0.0001) and implicit time (Fig. 9L) (r = 0.7150, *p* = 0.0019) for TG, all with high statistical significance.

Weak, statistically insignificant, correlations were found between mixed rod and cone ERG responses and blood glucose (Fig. S6A,D,G,J), TC (Fig. S6B,E,H,K), and TG (Fig. S6C,F,I,L). A moderate, statistically significant, negative correlation was seen between photopic a-wave implicit time (Fig. S7G) and blood glucose. Weak, negative correlations were seen between photopic a- and b-wave amplitudes (Fig. S7B,E) (r =  − 0.2949, *p* = 0.2860, and r = 0. − 3798, *p* = 0.1626, respectively) for TC. Furthermore, TG also displayed weak, negative correlations between photopic a- and b-wave amplitudes (Fig. S7C,F) (r =  − 0.3086, *p* = 0.2631, and r =  − 0.3787, *p* = 0.1640), but did not achieve statistical significance. Strong, negative correlations between photopic a-wave implicit time and TC (Fig. S7H) (r =  − 0.7272, *p* = 0.0014) and TG (Fig. S7I) (r =  − 0.7444, *p* = 0.0009) were found to be highly significant while negative correlations between photopic b-wave implicit time and TC (Fig. S7K) (r =  − 0.3498, *p* = 0.1842) and TG (Fig. S7L) (r = 0.4242, *p* = 0.1015) were weak to moderate, but not statistically significant. Our findings show that there is a relationship between blood glucose and elevation of total cholesterol and triglycerides that affect inner retinal function components of the ERG.

## Discussion

The present study has successfully demonstrated a mouse model in which mechanisms inherent to early-stage changes in retina function may be evaluated after experimentally induced dyslipidemia in the presence or absence of hyperglycemia. Using a well-accepted mouse model of T2DM in which lipids are minimally disturbed, but hyperglycemia is the predominant symptom (*db/db*), permits the evaluation of the effects of hyperglycemia alone on DR development. Similarly, use of the well-established P-407-induced mouse model of dyslipidemia, which does not perturb either blood glucose or plasma insulin levels^41^, allows for an assessment of how lipid derangements alone contribute to the development of DR. Importantly, the administration of P-407 to *db/db* mice makes possible the simultaneous presence of both disease mechanisms (i.e., hyperglycemia and dyslipidemia), which more closely resembles the clinical symptoms of DR experienced by most patients with T2DM and the metabolic syndrome. For the present study using P-407-treated *db/db* mice, we demonstrated that the effects of hyperglycemia and dyslipidemia were synergistic, rather than additive, in terms of early-stage changes in retina function, with the limitation that data result from the study of male mice only. Only male mice were included in this study to avoid the potential confounding effect of sex on our results, as sex differences with the metabolic syndrome have been observed in both humans and rodents^50–55^, although future studies using this novel model will include female mice.

While there have been several studies that suggest limited relation of serum lipids to the development of DR, there have also been studies that present evidence to the contrary. For example, it has been reported that TC and/or LDL levels can serve as predictors for the development of DR and hard exudates^56–58^. The link between elevated LDL levels and the development of DR may arise due to the toxic effects of oxLDL, which is generated in the P-407 model following oxidative modification of LDL^32^. Additionally, it is known that oxLDL is toxic to retinal capillary endothelial cells and pericytes in culture^59^ and oxLDL has been detected in the inner retina of humans preceding onset of clinically diagnosed DR^60^, in addition to Akita mice [a genetic model of T1DM]^61^.

In WT P-407 and *db/db* P-407 mice used in the present study, it is conceivable that the retinal microenvironment may contain reactive oxygen species (ROS) [e.g., alpha-oxygen, hydroxyl radical, peroxyl radical, etc*.*] and lipid peroxidation byproducts [e.g., malondialdehyde and 4-hydroxynonenal (4-HNE)], which could potentially lead to microvascular injury, disruption of neurovascular coupling, or oxidatively-mediated DNA damage^62–64^. However, it is important to point out that to date, we have only demonstrated the presence of lipid peroxidation byproducts such as malondialdehyde and 4-HNE in the plasma of P-407-treated wild type mice (i.e., WT P-407 mice)^32^. In fact, the presence of malondialdehyde in the plasma of WT P-407 mice gave rise to a significant increase in not only malondialdehyde-modified LDL (i.e., oxLDL), but also a significant increase in the plasma titers of immunoglobulin (Ig) G (T-cell-dependent) and IgM (T-cell-independent) autoantibodies against oxLDL^32^. This is noteworthy, given that lipid peroxidation is considerably higher in people with metabolic syndrome^65^, but also in DR patients especially^66^. It is well known that persistent lipid peroxidation produces a state of chronic inflammation. Thus, one of the unique advantages of the P-407-induced mouse model is that retinal neurodegeneration, initiated by the resultant toxic byproducts of lipid peroxidation, may be assessed in the context of dyslipidemia alone.

The above finding has enormous implications in the pathogenesis of DR, because similar to advanced glycation end-products (AGE’s) formed as a result of hyperglycemia, lipid peroxidation byproducts, arising from lipid peroxidation of polyunsaturated fatty acids, can form protein adducts and crosslinks known as advanced lipoxidation end-products (ALE’s), with both AGE’s and ALE’s producing adverse toxic effects (e.g., inflammation and oxidative stress) in the retinal microenvironment^62–64,67^.

An additional factor that may contribute to a retina microenvironment potentially enriched with ROS, oxidants, free radicals, and lipid peroxidation byproducts, is the drastic reduction in the HDL fraction of TC when P-407 is administered to either WT or *db/db* mice. A significant decrease in the HDL fraction for both WT P-407 (~ 30%) and *db/db* P-407 (~ 20%) mice in the present study means that there is not only reduced levels of HDL present, but also HDL that is ‘dysfunctional’ with regard to its antioxidant properties, since Yasuda and Johnston previously reported that HDL from WT P-407 mice was dysfunctional^31^. Taken together, the combination of oxLDL^32^ and HDL that has been rendered ‘dysfunctional’ with regard to its antioxidant properties (which are provided by the presence of apoA1, paraoxonase-1, and platelet-activating factor acetylhydrolase; n.b., there is approximately a 30% reduction in all three in WT P-407 mice^31^) may produce a toxic retinal microenvironment characterized by ‘oxidative stress’. Thus, we would suggest that a toxic retinal microenvironment produced by oxidized byproducts resulting from lipid peroxidation during sustained/chronic dyslipidemia (i.e., local effects) might possibly play a more important role in the advancement of DR than the elevated plasma concentration of any specific lipid or lipoprotein.

Triglycerides are generally associated with VLDL, and our model of combined hyperglycemia and dyslipidemia (*db/db* P-407) would support this association. That is, the effect of an equivalent dose of P-407 administered to *db/db* mice, relative to WT P-407 mice, resulted in a synergistic response (increase) in total TG. The highly significant (*p* < 0.001) increase in TG in *db/db* P-407 mice, relative to WT P-407 mice, most likely accounts for the profound expansion of the VLDL fraction of TC that was observed in *db/db* P-407 mice. Specifically, the LDL and VLDL fractions of TC in WT P-407 mice were approximately equal, whereas the VLDL fraction in the *db/db* P-407 mice, relative to the LDL fraction, was almost sevenfold greater, most likely as a means to accommodate the significant increase in TG observed in *db/db* P-407 mice. A greatly expanded VLDL fraction in *db/db* P-407 mice would appear to further support literature findings that the two groups of lipids in T2DM patients that are possibly the most problematic, independent risk factors giving rise to the development of DR are TG and VLDL^30^.

Our findings in the present study would strongly suggest that both dyslipidemia and hyperglycemia play crucial roles in the development of DR, with their detrimental effects on retinal function and associated vision loss. Accordingly, we now address our findings regarding the loss in VA and CS, as well as the various changes we observed in the ERGs, for the groups of mice used in the present study. It should be emphasized at the outset that an ERG is a particularly important aspect of diagnosis and therapeutic intervention for DR, since there is typically a significant amount of time separating the development of DR from the initial diagnosis of diabetes mellitus. Abnormalities in the ERG can appear at an early stage of the disease prior to any apparent changes in the fundus. Multiple ERG parameters can be impacted by DR. For example, with mild and moderate NPDR, these parameters include scotopic b-wave amplitude and implicit times, the amplitudes of oscillatory potentials (OP) evoked by all blue and white flashes (as well as selective decline of OP2 and OP3), OP implicit times, 30-Hz flicker amplitude and implicit times, and photopic ERG implicit times^68–71^. Additionally, reduced or absent OPs, decreased scotopic and photopic a- and b-waves, and delays in 30-Hz flicker implicit times have been reported with PDR^68,71–75^.

In the present study, and as it pertains to vision and retinal function, *db/db* P-407 mice were determined to have both pronounced loss of retinal function and vision loss when the effects of either dyslipidemia alone, or hyperglycemia alone, were not yet evident. More specifically, the *db/db* P-407 group of mice exhibited reduced VA and CS relative to WT P-407 (dyslipidemia only) and *db/db* (hyperglycemia only) mice. Additionally, we observed abnormalities in scotopic a- and b-wave amplitudes, scotopic a-wave implicit times, mixed rods and cones a- and b-wave amplitudes and implicit times, isolated photopic b-wave amplitudes, and photopic a-wave implicit times; all, of which, contributed to the changes in the ERGs determined for *db/db* diabetic mice subjected to sustained (1 month) P-407-induced dyslipidemia (i.e.*, db/db* P-407 mice). Of particular note, our findings would appear to show a decrease in retinal function based on scotopic, mixed rods and cones, and photopic amplitudes of a- and b-waves, together with prolonged implicit times of the ERG stimulus for scotopic a-wave, mixed rods and cones, and isolated photopic responses in *db/db* P-407 mice. In summary, based on our findings that TC and TG were strongly and significantly correlated with reduced VA, and moderately, but not significantly, correlated with reduced CS, together with our findings that there was a strong, very-significant correlation of both TC and TG with ERG scotopic b-wave amplitude and implicit time, it would seem to support our assertion that a dyslipidemic environment significantly contributes to a decline in retinal function, vision loss, and the development of DR in diabetic (*db/db* P-407) mice.

It would certainly seem worthwhile to propose possible underlying mechanisms that may be associated with the decline in retinal function, vision loss, and development of early-stage DR that we observed in our novel mouse model (*db/db* P-407) of simultaneous dyslipidemia (with the presence of oxLDL, 4-HNE, and ‘dysfunctional’ HDL) and hyperglycemia. To this end, and in view of, (1) the specific ERG changes we observed in *db/db* P-407 mice, and (2) the significant role that ‘oxidative stress’ plays in the development of DR, we now turn our attention to the neurovascular unit. Briefly, the neurovascular unit is comprised of the neural unit [retinal ganglion cells (RGCs) and glial cells (microglia, Müller cells, and astrocytes)] and the vascular unit [endothelial cells (ECs) and pericytes]^76^. The ECs and pericytes, together with Müller cells and astrocytes, form the inner blood-retina barrier^76^. Importantly, about 90% of the retinal glia are Müller cells, and due to the fact that they traverse the boundaries of the neuroretina from the outer limiting membrane to the inner, Müller cells play a predominant role in physiological and pathological processes and function in a symbiotic relationship between the retina neurons and vasculature^77–80^.

As mentioned above, based on the specific ERG changes we observed for *db/db* P-407 mice, we would suggest, although we have no unequivocal direct evidence, that Müller cell dysfunction is the principal mechanism responsible for retina impairment in early-stage DR. Moreover, due to the ‘oxidative stress’ created by P-407-induced dyslipidemia, as well as hyperglycemia, and based on the fact that ‘oxidative stress’ makes a substantial contribution towards the loss of vulnerable neurons like RGCs^81^, we would also suggest that the decrease in retinal function we determined in *db/db* P-407 mice was further exacerbated by death of RGCs. After all, Lin et al. suggested that ‘oxidative stress’ triggers the process of autophagy in RGCs^82^, and suppression of ‘oxidative stress’ in vivo decreases RGC apoptosis in *db/db* mice^83^. Taken together, we propose that the combination of dyslipidemia (potentially creating a retinal microenvironment of ‘oxidative stress’) and hyperglycemia adversely impacts the function and/or survival of Müller cells and RGCs in the *db/db* P-407 mouse model, resulting in a decline in retinal function, vision loss, and the development of early-stage DR.

## Conclusion

In conclusion, the P-407 mouse model of dyslipidemia specifically elevates TG and VLDL, both of which are independent risk factors for DR in humans^30^. Additional future studies with the *db/db* P-407 mouse model will explore the dual role that Müller cells play in the development of DR, especially as it pertains to the dysfunction of specific growth factors (e.g., bFGF and VEGF) during a state of dyslipidemia and hyperglycemia, and how this interplay contributes to a decline in retinal function, vision loss, BRB breakdown, and the development of early-stage DR. The findings in the present study will allow vision researchers to mechanistically dissect the contributions of dyslipidemia alone (P-407 model), hyperglycemia alone (*db/db* model), and both disease-generating processes simultaneously (*db/db* P-407) to more completely elucidate the pathogenesis of DR, as well as reveal potential therapeutic targets in an effort to either slow, or halt, its progression.

### Supplementary Information

Supplementary Information 1.

Supplementary Information 2.

Supplementary Information 3.

Supplementary Information 4.

Supplementary Information 5.

Supplementary Information 6.

Supplementary Information 7.

Supplementary Information 8.

Supplementary Information 9.

Supplementary Information 10.

## Data Availability

The datasets used and analyzed during the current study are available from the corresponding author on reasonable request.
