# Supplementary material for: Synergism of mechanisms underlying early-stage changes in retina function in male hyperglycemic db/db mice in the absence and presence of chemically-induced dyslipidemia
Source: Sci Rep. 2023 Oct 13;13:17347. doi: 10.1038/s41598-023-44446-3 (PMC10576038; doi:10.1038/s41598-023-44446-3)
Supplement: Supplementary file 1 — Supplementary Information 1. [file 41598_2023_44446_MOESM1_ESM.pdf]

Supplemental Figure Legend

Supplemental Figure 1: Retinal function in experimentally induced dyslipidemia and hyperglycemia on mixed rod and cone and photopic ERG responses. Full-field flash ERG for mixed rod and cone amplitudes (S1A and C), implicit times (S1B and D), and photopic amplitudes (S1F and H) and implicit times (S1G and I) were measured from 8-week-old male WT and *db/db* mice divided into four cohorts total. WT controls (n = 10) are indicated with circles, WT P-407 (n = 7) with triangles, *db/db* (n = 7) with squares, and *db/db* P-407 (n = 6) with inverted triangles. A representative trace from a single eye out of each group is presented in (S1E and J). Solid black and red lines indicate WT animals and *db/db* animals respectively, while dashed lines indicate animals treated with P-407. Data are expressed as mean ± SEM. One-way ANOVA with Tukey's post-test was used to determine statistical significance. * and ** designate a statistically significant difference ($p < 0.05$ and $p < 0.01$ respectively) between mean values.

Supplemental Figure 2: Effect of experimentally induced dyslipidemia and hyperglycemia on scotopic and photopic ERG flashes. Scotopic amplitude (S2A and C) and implicit time (S2B and D) were measured from responses to flashes beginning at −5.5 to 1.0 log cd·s/m^2 and -2.5 to 1.0 log cd·s/m^2 for photopic amplitude (S2E and G) and implicit time (S2F and H) of 8-week-old male WT and *db/db* mice divided into 4 cohorts. WT controls (n = 10) are symbolized with circles, WT P-407 (n = 7) with triangles, *db/db* (n = 7) with squares, and *db/db* P-407 (n = 6) with inverted triangles. Solid black and red lines indicate WT animals and *db/db* animals respectively, while dashed lines indicate animals treated with P-407. Data are expressed as mean ± SEM. One-way ANOVA with Tukey's post-test was used to determine statistical significance and is shown in Supplemental Table S2.

Supplemental Figure 3: Threshold responses in dark-adapted and light-adapted ERG. The pSTR (S3A), nSTR (S3B), STR amplitude (S3C), STR amplitude implicit time (S3D), PhNR amplitude (S3E), and PhNR implicit time (S3F) were measured from responses to flashes below −4.5 to −3.5 log cd·s/m^2 for scotopic and 0 to 1.5 log cd·s/m^2 for photopic responses of 8-week old male WT and *db/db* mice divided into 4 cohorts. WT controls (n = 10) are symbolized with circles, WT P-407 (n = 7) with triangles, *db/db* (n = 7) with squares, and *db/db* P-407 (n = 6) with inverted triangles. Solid black and red lines indicate WT animals and *db/db* animals respectively, while dashed lines indicate animals treated with P-407. Data are expressed as mean ± SEM. A mixed effect analysis with Tukey's post-test was used to determine statistical significance and values are listed directly in panels where identified.

Supplemental Figure 4: Relationship between mixed rod and cone ERG function and behavior assessment of optomotor reflex. Correlations were determined between mixed rod and cone ERG function and visual acuity (S4A, C, E, and G) and contrast sensitivity (S4B, D, F, and H). WT controls (n = 10) are symbolized with circles, WT P-407 (n = 7) with triangles, *db/db* (n = 7) with squares, and *db/db* P-407 (n = 6) with inverted triangles. Dashed lines around the regression line indicate 95% CI. Specific Pearson correlation coefficient r, respective p-values, and coefficient of determination R^2 are listed directly in panels.

Supplemental Figure 5: Relationship between photopic ERG function and behavior assessment of optomotor reflex. Correlations were determined between photopic ERG function and visual acuity (S5A, C, E, and G) and contrast sensitivity (S5B, D, F, and H). WT controls (n = 10) are symbolized with circles, WT P-407 (n = 7) with triangles, *db/db* (n = 7) with squares, and *db/db* P-407 (n = 6) with inverted triangles. Dashed lines around the regression line indicate 95% CI. Specific Pearson correlation coefficient r, respective p-values, and coefficient of determination R^2 are listed directly in panels.

Supplemental Figure 6: Relationship between mixed rod and cone ERG function and blood glucose and plasma lipid levels. Correlations were determined between mixed rod and cone a- and b-wave amplitudes, implicit time, and blood glucose (S6A, D, G, and J), total cholesterol (S6B, E, H, and K), and plasma triglycerides (S6C, F, I, and L). WT controls (n = 10) are symbolized with circles, WT P-407 (n = 7) with triangles, *db/db* (n = 7) with squares, and *db/db* P-407 (n = 6) with inverted triangles. Dashed lines around the regression line indicate 95% CI. Specific Pearson correlation coefficient r, respective p-values, and coefficient of determination R^2 are listed directly in panels.

Supplemental Figure 7: Relationship between photopic ERG function and blood glucose and plasma lipid levels. Correlations were determined between photopic a- and b-wave amplitudes, implicit time, and blood glucose (S7A, D, G, and J), total cholesterol (S7B, E, H, and K), and plasma triglycerides (S7C, F, I, and L). WT controls (n = 10) are symbolized with circles, WT P-407 (n = 7) with triangles, *db/db* (n = 7) with squares, and *db/db* P-407 (n = 6) with inverted triangles. Dashed lines around the regression line indicate 95% CI. Specific Pearson correlation coefficient r, respective p-values, and coefficient of determination R^2 are listed directly in panels.
