# Supplementary material for: Synergism of mechanisms underlying early-stage changes in retina function in male hyperglycemic db/db mice in the absence and presence of chemically-induced dyslipidemia
Source: Sci Rep. 2023 Oct 13;13:17347. doi: 10.1038/s41598-023-44446-3 (PMC10576038; doi:10.1038/s41598-023-44446-3)
Supplement: Supplementary file 9 — Supplementary Information 9. [file 41598_2023_44446_MOESM9_ESM.pdf]

Supplemental Tables

Supplemental Table S1 to Figure 3B: Lipoprotein Fractions Relative to Total Cholesterol: The Absence or Presence of Equivalent P-407-induced Dyslipidemia in Control (WT) and Diabetes Spontaneous Mutation (*db/db*) Mice

Group	[1] HDL (%)	[2] LDL (%)	[3] VLDL (%)
**WT**	79.4 ± 6.3	14.2 ± 1.1	5.4 ± 2.6
**WT P-407**	[a] 53.5 ± 4.1	[b] 20.5 ± 2.3	[a] 25.6 ± 7.1
db/db	68.8 ± 8.7	10.6 ± 2.9	20.6 ± 3.4
***db/db* P-407**	[c] 49.0 ± 5.5	6.5 ± 1.8	[d] 44.0 ± 6.2

[1]Mean HDL concentration as a percent of the mean TC concentration

[2]Mean LDL concentration as a percent of the mean TC concentration

[3]Mean VLDL concentration as a percent of the mean TC concentration

[a]$p < 0.01$ compared to the mean value for WT

[b]$p < 0.05$ compared to the mean value for WT

[c]$p < 0.05$ compared to the mean value for *db/db*

[d]$p < 0.01$ compared to the mean value for *db/db*

Supplemental Table S2: Scotopic and Photopic Electroretinogram Flash Intensity Values of Amplitude and Latency for P-407-induced Dyslipidemia in Control (WT) and Genetically-Modified (*db/db*) Mice
