# Supplementary material for: Synergism of mechanisms underlying early-stage changes in retina function in male hyperglycemic db/db mice in the absence and presence of chemically-induced dyslipidemia
Source: Sci Rep. 2023 Oct 13;13:17347. doi: 10.1038/s41598-023-44446-3 (PMC10576038; doi:10.1038/s41598-023-44446-3)
Supplement: Supplementary file 10 — Supplementary Information 10. [file 41598_2023_44446_MOESM10_ESM.pdf]

Supplemental Table S2. Electroretinogram Flash Intensity Values of Amplitude and Latency for P-407-induced Dyslipidemia in Control (WT) and Genetically-Modified (*db/db*) Mice

Scotopic a-wave Amplitude

Flash Intensity	WT			WT P-407				*db/db*				*db/db* P-407			
Log mcd.s/m^2	Mean	SEM	n	Mean	SEM	p value	n	Mean	SEM	p value	n	Mean	SEM	p value	n
-5.5	5.54	1.35	10	17.98	3.37	0.007	10	13.19	2.15	0.012	14	23.54	8.09	ns	12
-5.0	5.16	1.37	10	21.11	4.80	0.012	10	17.64	4.88	0.047	14	24.78	8.01	0.039	12
-4.5	6.52	1.50	10	20.81	5.04	0.029	10	19.78	6.46	ns	14	29.40	7.96	0.018	12
-4.0	12.00	3.00	10	39.51	8.22	0.012	10	37.20	8.98	0.032	14	32.32	6.57	0.016	12
-3.5	19.55	7.98	10	81.47	14.96	0.004	10	73.39	10.68	0.001	14	55.81	12.63	0.031	12
-3.0	11.61	2.63	10	66.81	11.64	p<.001	10	70.55	21.94	0.035	14	46.23	7.47	p<.001	12
-2.5	19.28	4.75	10	81.44	12.15	p<.001	10	73.94	13.54	0.003	14	58.03	8.68	0.001	12
-2.5	24.39	5.67	10	62.56	10.69	0.010	10	67.41	6.42	p<.001	14	36.80	9.25	ns	12
-2.0	44.21	4.96	10	51.28	7.83	ns	10	80.67	16.50	ns	14	55.06	11.07	ns	12
-2.0	96.15	6.62	10	102.31	9.11	ns	10	74.29	8.40	ns	14	63.78	7.10	0.004	12
-1.5	177.03	7.43	10	165.35	7.91	ns	10	188.29	14.32	ns	14	106.61	15.34	0.001	12
-1.0	242.02	24.08	10	222.37	18.11	ns	10	259.37	14.39	ns	14	178.58	17.56	0.044	12
-0.5	286.19	9.66	10	237.71	15.80	0.027	10	285.27	19.83	ns	14	229.18	21.21	0.033	12
0.0	343.60	13.90	10	258.61	18.49	0.002	10	322.32	33.73	ns	14	258.60	21.67	0.005	12
0.5	383.76	19.24	10	349.56	36.52	ns	10	569.41	141.75	ns	14	354.65	31.97	ns	12
1.0	408.55	26.89	10	364.15	36.26	ns	10	703.19	161.23	ns	14	341.83	43.67	ns	12

Scotopic a-wave Latency

Flash Intensity	WT		WT P-407			*db/db*			*db/db* P-407		
Log mcd.s/m^2	Mean	SEM	Mean	SEM	p value	Mean	SEM	p value	Mean	SEM	p value
-5.5	36.63	11.50	14.84	8.47	ns	13.99	6.71	ns	4.76	3.87	0.010
-5.0	36.57	12.82	17.01	6.64	ns	19.86	6.33	ns	4.57	4.37	0.019
-4.5	23.77	7.74	12.32	4.93	ns	14.34	5.63	ns	21.66	12.10	ns
-4.0	23.17	5.52	16.09	4.94	ns	17.54	5.75	ns	23.20	9.34	ns
-3.5	25.98	6.71	29.19	7.07	ns	28.67	5.70	ns	23.16	6.45	ns
-3.0	36.21	6.64	21.60	5.80	ns	18.69	5.68	ns	12.06	5.26	0.009
-2.5	26.89	5.22	22.66	5.33	ns	21.06	5.11	ns	16.66	6.12	ns
-2.5	38.23	1.49	39.15	6.80	ns	34.42	5.07	ns	39.50	8.11	ns
-2.0	45.71	1.02	33.37	7.82	ns	33.26	4.77	0.041	12.90	5.22	p<.001
-2.0	37.76	1.05	39.56	4.29	ns	36.11	3.73	ns	52.27	10.14	ns
-1.5	30.21	1.71	32.29	2.31	ns	34.84	1.90	ns	33.07	3.60	ns
-1.0	25.87	2.91	30.99	1.48	ns	31.57	1.70	ns	37.91	5.37	ns
-0.5	21.06	1.10	21.66	1.50	ns	20.69	0.75	ns	23.34	1.47	ns
0.0	14.38	0.17	16.07	1.14	ns	25.28	8.44	ns	17.36	0.99	0.014
0.5	11.33	1.08	21.24	6.18	ns	13.65	1.38	ns	16.68	2.18	ns
1.0	9.52	0.86	21.87	7.99	ns	9.44	1.52	ns	16.23	6.12	ns

Scotopic b-wave Amplitude

Flash Intensity	WT		WT P-407			*db/db*			*db/db* P-407		
Log mcd.s/m^2	Mean	SEM	Mean	SEM	p value	Mean	SEM	p value	Mean	SEM	p value
-5.5	15.29	1.53	36.22	4.16	p<.001	29.36	3.25	0.002	33.45	9.10	ns
-5.0	20.70	1.05	40.90	4.53	0.001	33.82	5.07	0.043	38.52	8.79	ns
-4.5	37.37	1.54	50.26	4.07	0.017	49.95	7.26	ns	47.84	7.32	ns
-4.0	79.53	3.01	102.68	10.06	ns	109.77	11.20	ns	66.82	6.49	ns
-3.5	131.68	8.43	180.98	16.72	0.029	212.41	16.45	p<.001	131.79	13.90	ns
-3.0	194.47	7.07	230.08	15.61	ns	255.00	28.11	ns	158.68	12.97	0.033
-2.5	292.68	8.84	283.16	16.53	ns	341.98	20.63	ns	201.21	19.27	p<.001
-2.5	359.73	12.10	256.63	16.70	p<.001	291.10	24.34	0.036	168.16	19.74	p<.001
-2.0	332.44	11.59	259.23	23.04	0.020	357.33	20.70	ns	216.38	19.03	p<.001
-2.0	472.50	9.61	346.24	26.97	0.001	384.39	41.00	ns	234.53	35.18	p<.001
-1.5	565.68	20.46	383.12	28.36	p<.001	494.48	41.00	ns	242.37	54.64	p<.001
-1.0	737.35	67.74	438.60	40.59	p<.001	540.60	45.76	0.020	318.66	48.99	p<.001
-0.5	667.66	30.86	439.49	35.67	p<.001	571.79	45.37	ns	334.88	51.36	p<.001
0.0	706.88	26.87	445.99	40.59	p<.001	585.39	67.58	ns	366.56	51.88	p<.001
0.5	906.04	75.48	558.13	53.60	p<.001	890.06	145.07	ns	478.43	57.71	p<.001
1.0	940.95	54.52	585.20	43.01	p<.001	1120.8	202.85	ns	493.09	67.27	p<.001

Scotopic b-wave Latency

Flash Intensity	WT		WT P-407			*db/db*			*db/db* P-407		
Log mcd.s/m^2	Mean	SEM	Mean	SEM	p value	Mean	SEM	p value	Mean	SEM	p value
-5.5	119.43	10.48	125.63	9.90	ns	151.73	6.58	ns	127.57	5.93	ns
-5.0	137.67	3.89	146.90	5.38	ns	129.56	5.16	ns	138.32	3.43	ns
-4.5	137.85	2.98	128.74	5.05	ns	126.74	2.98	ns	145.79	8.83	ns
-4.0	134.81	2.96	127.41	3.95	ns	130.77	6.25	ns	131.70	3.79	ns
-3.5	127.67	3.68	117.81	6.00	ns	118.01	5.34	ns	129.03	5.61	ns
-3.0	121.44	5.83	112.46	5.67	ns	101.76	4.78	ns	127.39	5.18	ns
-2.5	115.73	6.93	101.12	5.67	ns	84.37	6.13	ns	123.85	4.42	ns
-2.5	74.86	7.79	94.36	6.32	ns	74.99	3.91	0.005	104.83	7.62	0.0129
-2.0	101.32	8.51	89.98	3.01	ns	66.27	4.88	ns	98.68	6.18	ns
-2.0	54.86	1.20	73.12	4.87	0.005	53.17	2.95	ns	71.54	5.63	0.0153
-1.5	44.11	4.93	53.43	2.14	ns	49.65	2.66	ns	75.67	11.78	0.0322
-1.0	50.16	4.54	49.75	2.01	ns	47.37	2.56	ns	67.47	15.65	ns
-0.5	44.10	0.99	55.63	6.52	ns	46.14	2.44	ns	66.79	10.75	ns
0.0	41.63	0.73	47.45	1.96	0.024	47.18	2.37	0.044	72.75	12.92	0.0408
0.5	38.45	3.52	55.33	6.00	0.040	49.11	5.02	ns	76.65	12.05	0.0110
1.0	52.19	7.84	49.22	4.14	ns	49.11	5.02	ns	61.84	10.69	ns

Photopic a-wave Amplitude

Flash Intensity	WT			WT P-407				*db/db*				*db/db* P-407			
Log mcd.s/m^2	Mean	SEM	n	Mean	SEM	p value	n	Mean	SEM	p value	n	Mean	SEM	p value	n
-2.5	9.04	2.13	10	15.20	2.63	ns	10	24.09	7.78	ns	14	30.89	7.31	0.016	12
-2.0	41.74	24.82	10	18.28	2.23	ns	10	60.97	41.35	ns	14	18.23	3.32	ns	12
-1.5	11.71	4.61	10	21.29	4.20	ns	10	48.78	22.19	ns	14	13.28	2.92	ns	12
-1.0	58.50	45.95	10	16.37	3.36	ns	10	44.10	23.02	ns	14	21.23	5.48	ns	12
-0.5	47.04	31.53	10	24.72	3.55	ns	10	92.49	50.09	ns	14	11.46	1.61	ns	12
0.0	68.43	34.40	10	27.41	4.00	ns	10	36.10	13.58	ns	14	30.48	9.14	ns	12
0.5	46.48	18.72	10	32.20	3.41	ns	10	69.05	23.72	ns	14	35.95	8.81	ns	12
1.0	123.95	64.23	10	33.96	5.96	ns	10	69.05	23.72	ns	14	89.28	49.23	ns	12

Photopic a-wave Latency

Flash Intensity	WT		WT P-407			*db/db*			*db/db* P-407		
Log mcd.s/m^2	Mean	SEM	Mean	SEM	p value	Mean	SEM	p value	Mean	SEM	p value
-2.5	44.86	13.48	34.67	6.92	ns	29.93	8.35	ns	28.83	9.05	ns
-2.0	33.27	9.34	45.10	9.87	ns	22.59	7.30	ns	31.72	10.84	ns
-1.5	28.63	8.52	27.99	7.01	ns	26.81	5.49	ns	33.20	8.41	ns
-1.0	28.87	5.34	26.86	3.42	ns	28.39	7.89	ns	25.00	2.24	ns
-0.5	24.89	2.03	30.01	4.04	ns	18.85	3.44	ns	-19.25	22.82	ns
0.0	21.05	4.07	31.41	4.41	ns	20.37	3.65	ns	23.56	2.94	ns
0.5	17.33	2.74	32.59	3.33	0.003	14.99	2.46	ns	16.52	4.01	ns
1.0	15.52	2.64	28.32	3.55	0.014	14.99	2.46	ns	15.01	3.71	ns

Photopic b-wave Amplitude

Flash Intensity	WT		WT P-407			*db/db*			*db/db* P-407		
Log mcd.s/m^2	Mean	SEM	Mean	SEM	p value	Mean	SEM	p value	Mean	SEM	p value
-2.5	51.19	34.56	33.53	4.56	ns	72.25	24.92	ns	46.96	10.75	ns
-2.0	86.60	57.63	34.89	4.58	ns	113.18	55.36	ns	50.07	10.54	ns
-1.5	46.69	21.87	35.57	4.36	ns	97.59	43.84	ns	36.03	6.93	ns
-1.0	71.45	41.57	36.82	4.50	ns	120.65	42.99	ns	35.89	6.32	ns
-0.5	105.03	50.37	54.73	5.30	ns	224.06	84.80	ns	42.23	4.66	ns
0.0	182.75	61.28	74.26	6.28	0.048	174.49	21.11	ns	79.02	11.92	ns
0.5	208.29	54.13	94.82	9.71	0.024	234.71	43.28	ns	117.54	16.84	ns
1.0	307.48	89.92	113.54	16.99	0.021	234.71	43.28	ns	183.79	52.28	ns

Photopic b-wave Latency

Flash Intensity	WT		WT P-407			*db/db*			*db/db* P-407		
Log mcd.s/m^2	Mean	SEM	Mean	SEM	p value	Mean	SEM	p value	Mean	SEM	p value
-2.5	111.53	14.75	87.98	13.48	ns	82.70	12.64	ns	83.42	14.75	ns
-2.0	69.77	15.94	78.01	9.34	ns	59.52	11.64	ns	81.98	13.82	ns
-1.5	64.86	12.13	49.44	9.26	ns	65.10	9.21	ns	82.42	11.43	ns
-1.0	50.60	9.09	57.53	6.51	ns	54.21	8.73	ns	63.12	6.23	ns
-0.5	36.11	4.69	57.34	4.98	0.007	47.65	4.02	ns	48.46	6.26	ns
0.0	40.52	8.87	60.69	7.51	ns	56.55	6.05	ns	52.55	6.34	ns
0.5	44.57	4.77	60.24	2.58	0.005	46.61	5.66	ns	54.71	11.33	ns
1.0	41.11	6.06	57.44	5.01	0.049	46.61	5.66	ns	41.04	8.35	ns
